# Low Levels of the Reverse Transactivator Fail to Induce Target Transgene Expression in Vascular Smooth Muscle Cells

**DOI:** 10.1371/journal.pone.0104098

**Published:** 2014-08-04

**Authors:** Nikenza Viceconte, Tomás McKenna, Maria Eriksson

**Affiliations:** Department of Biosciences and Nutrition, Center for Biosciences, Karolinska Institutet, Novum, Huddinge, Sweden; Faculty of Animal Sciences and Food Engineering, University of São Paulo, Pirassununga, SP, Brazil, Brazil

## Abstract

Hutchinson-Gilford progeria syndrome (HGPS) is a genetic disease with multiple features that are suggestive of premature aging. Most patients with HGPS carry a mutation on one of their copies of the *LMNA* gene. The *LMNA* gene encodes the lamin A and lamin C proteins, which are the major proteins of the nuclear lamina. The organs of the cardiovascular system are amongst those that are most severely affected in HGPS, undergoing a progressive depletion of vascular smooth muscle cells, and most children with HGPS die in their early teens from cardio-vascular disease and other complications from atherosclerosis. In this study, we developed a transgenic mouse model based on the tet-ON system to increase the understanding of the molecular mechanisms leading to the most lethal aspect of HGPS. To induce the expression of the most common HGPS mutation, *LMNA* c.1824C>T; p.G608G, in the vascular smooth muscle cells of the aortic arch and thoracic aorta, we used the previously described reverse tetracycline-controlled transactivator, sm22α-rtTA. However, the expression of the reverse sm22α-transactivator was barely detectable in the arteries, and this low level of expression was not sufficient to induce the expression of the target human lamin A minigene. The results from this study are important because they suggest caution during the use of previously functional transgenic animal models and emphasize the importance of assessing transgene expression over time.

## Introduction

Hutchinson-Gilford progeria syndrome (HGPS) is a very rare genetic disorder characterized by multiple features and pathologies typical of advanced age. Initial symptoms start to appear approximately one year after birth and include postnatal growth retardation, decreased subcutaneous fat, alopecia and failure to thrive [1], [2]. The disorder is usually caused by a *de novo* point mutation c.1824C>T, p.G608G in exon 11 of the *LMNA* gene. The *LMNA* gene encodes the lamin A and lamin C proteins, which are the major proteins of the nuclear lamina located beneath the inner nuclear membrane [3].

The *LMNA* c.1824C>T, p.G608G mutation and other less-prevalent mutations, including the *LMNA* c.1822G>A, p.G608S mutation [3], [4], result in the partial activation of a cryptic splice site and in the removal of the 150 carboxy-terminal nucleotides of exon 11 [3], [4]. This internal deletion leads to the expression of a truncated lamin A protein with an internal deletion of 50 amino acids, called progerin [3], [5], [6]. Since none of the mutations create a sequence with a perfect match to a splice site the full-length lamin A protein will also be expressed from the mutant allele. The accumulation of progerin is thought to affect nuclear structure and function, which in turn leads to the process of premature aging [5], [6]. Previous studies have shown that the cardiovascular phenotype in HGPS involves the progressive medial loss of vascular smooth muscle cells and replacement with proteoglycans and collagens [7], [8]. Further studies should be performed to explore the molecular dysfunction and activation of signaling pathways caused by progerin accumulation, particularly in the cardiovascular system, because such accumulation leads to death from myocardial infarction and/or stroke, usually during the second decade of life [1], [9].

Several mouse models have been developed to explore the functions of the lamin gene in health and disease [10]. One of the most commonly used inducible protein expression systems is regulated by tetracycline and its derivatives [11]. This system has regulatory and responsive element components. The regulatory component consists of one of the transactivators, tTA or rtTA, that is associated with a tissue specific promoter. The responsive element contains the target gene downstream of the *tetO* sequence (tetop). Various Tet-regulated expression systems have been developed to enable the induction or repression of the transcription of a target gene in response to tetracycline, Tet, or a derivative of tetracycline, usually doxycycline. These tet-ON or tet-OFF expression systems allow for the control of target gene expression in a specific tissue at different time points and are reversible [11], [12]. Several mouse models have been developed to study the underlying causes of HGPS [10], and two animal models are currently available to study the cardiovascular pathology of HGPS [8], [13]; however, none of these models is inducible or tissue-specific. Our goal was to induce the pathology of HGPS in arteries by developing an inducible, tissue-specific expression system based on the tet-ON system [11], under the control of the vascular smooth muscle cell specific promoter (sm22α) that is expressed during mouse embryogenesis (from embryonic day 9.5) and continuously active in postnatal vascular smooth muscle cells [14]–[15]. Here, we report one of the problems with the tet-ON system and emphasize the need to assess the expression of transgenes over time.

## Materials and Methods

### Ethics Statement

This study was performed in accordance with the guidelines for working with experimental animals set by the Karolinska Institute and all efforts were made to minimize animal suffering. All animal studies were approved by the Stockholm South Ethical review board (Dnr. S101–12 to Maria Eriksson).

### Transgenic animals

Transgenic mice were housed in a pathogen-free animal facility within the Karolinska University Hospital, Huddinge, Sweden, and maintained in a 12-hour light/dark cycle, at 20–22°C temperature and 50–65% air humidity. Heterozygous animals carrying the tissue-specific promoter-regulated reverse transactivator sm22α-rtTA (on the C57BL/6J genetic background) [15] were crossed with heterozygous tetop-LA^G608G^ animals (on the C57BL/6J or FVB/NCrl genetic background) carrying the human lamin A minigene containing the most common HGPS mutation, *LMNA* c.1824c>T; p.G608G (tetop-LA^G608G^; F1-line VF1–07) [16]. PCR genotyping was performed according to previously described procedures [15], [16]. Offspring that were positive for both transgenes, tetop-LA^G608G+^; sm22α-rtTA^+^, were referred to as bi-transgenic animals. The single transgenic offspring, tetop-LA^G608G+^; sm22α-rtTA^−^, were referred to as control mice. The non-transgenic offspring, tetop-LA^G608G−^; sm22α-rtTA^−^, were referred to as wild-type mice. The animals were given drinking water supplemented with 5% sucrose and doxycycline. A total of twenty-one litters were treated with doxycycline during embryogenesis, out of these one litter was given 1.5 mg/ml doxycycline and the remainder were given 2 mg/ml doxycycline. Sixteen litters were given 2 mg/ml doxycycline postnatally. Tissues from tetop-LA^G608G+^; NSE-tTA^+^, tetop-LA^G608G−^; NSE-tTA^−^, tetop-LA^G608G−^; NSE-tTA^+^ mice, and tetop-LA^G608G+^; Sp7-tTA^+^ [17], [18] were used as a controls.

### RNA extraction and RT-PCR analysis

Animals were sacrificed by administering an overdose of isoflurane (Baxter, Deerfield, IL, USA) at postnatal week 4 or 12, and the aortic arch, thoracic aorta and abdominal aorta were collected for analysis. RNA was extracted using the TriZol Reagent (Invitrogen, Carlsbad, CA, USA). Following RNA extraction, the samples were treated with DNase (Promega, Madison, WI, USA) and column purified (Qiagen, Valencia, CA, USA). The cDNA synthesis was performed on 0.5 µg total RNA with random hexamers using the SuperScript II Reverse Transcriptase kit (Invitrogen, Carlsbad, CA, USA), according to the manufacturer’s instructions. The expressions of transgenic human lamin A and lamin Adel150 (progerin) were analyzed by PCR using the primers 5′-AGTTCTGGGGGCTCTGGGT-3′, 5′-ACTGCAGCAGCTCGGGG-3′, and 5′-TCTGGGGGCTCTGGGC-3′ [16]. The fragment sizes for human lamin A and human lamin Adel150 were 276 and 123 base pairs, respectively [16]. PCR amplification of the cDNA using primers for β-actin (5′- CCTAGGCACCAGGGTGTGAT-3′ and 5′-CCATGTCGTCCCAGTTGGTAA-3′) was performed on all samples as a control. The expression of the transactivator rtTA was analyzed using the following primers: 5′-GACGCGCTAGACGATTTCGA-3′ and 5′-GGAAAGACCCCTAGGAATGC-3′[15].

### Protein extraction and Western blot

Protein was extracted from arteries of bi-transgenic and single transgenic animals as previously described [16]. To be able to obtain enough protein for the Western blot, tissue samples from aortic arch, thoracic aorta and abdominal aorta from each animal were pooled for protein extraction. Protein extracts from HGPS patient cell line AG11513A (Coriell Cell Respositories), tetop-LA^G608G−^; NSE-tTA^−^, tetop-LA^G608G+^; NSE-tTA^+^ and tetop-LA^G608G−^; NSE-tTA^+^ mice [17] were used as a controls. Enhanced protein separation Western blot analysis was performed using the Odyssey system (LI-COR Biosciences) and as previously described [16]. Primary antibodies used for Western blot were: anti-TetR (9G9, Clontech Laboratories Inc.), anti-TetR (631108, Clontech Laboratories Inc.), anti-human lamin A/C (JoL2, Chemicon), and anti-Β-actin (A5441, Sigma).

### Immunofluorescence, immunohistochemistry and imaging

Animals were sacrificed by administering an overdose of isoflurane and the aortic arch, thoracic aorta and abdominal aorta were collected from animals at postnatal weeks 4 or 12 from 5 wild-type and 5 bi-transgenic mice. The samples were fixed in 4% paraformaldehyde (pH 7.4) overnight and then dehydrated in 70% ethanol. After fixation, the tissues samples were embedded in paraffin, cut into 4-µm sections and dried at 60°C for 30 minutes. The sections were then stained with haematoxylin and eosin (H&E) according to standard procedures. For immunostaining analysis, the sections were re-hydrated, followed by antigen retrieval using a pressure cooker. The primary antibodies used were anti-human-lamin A/C (1∶30, JoL2, mouse, Chemicon) (cross reacts with lamin A/C and progerin of human origin [16]), anti-human-progerin (1∶100, 13A4, mouse, Enzo Life Science) (cross reacts with progerin of human origin [19], anti-human-lamin A/C (1∶75, N-18, goat, Santa Cruz) (cross reacts with lamin A/C and progerin of human origin and lamin A/C of mouse origin [16]), and anti-sm α actin conjugated to Cy3 (1∶200, 1A4, mouse, Sigma). The samples were incubated with the primary antibodies overnight at 4°C. The secondary antibodies used were Alexa 555-conjugated goat anti-mouse (1∶100, A-2122, Life Technologies) and Alexa 488-conjugated donkey anti-goat (1∶100, A-11055, Life Technologies). Blocking was performed with normalized goat or donkey serum, BSA or mouse-to-mouse blocking reagent (Scytek, Logan, UT, USA). The sections were mounted in Vectashield mounting media containing DAPI (Vector laboratories, Burlingame, CA, USA). Sections from aortic arch from tetop-LA^G608G+^; NSE-tTA^+^ [17] were used as controls. Imaging was performed using a Nikon A1+ imaging system, (Nikon Corporation, Japan), and images were analyzed using NIS elements (Nikon Corporation, Japan). Immunohistochemistry using the anti-Cleaved Caspase 3 (1∶200, Asp 175, Cell Signaling) primary antibody was performed on deparaffinized sections. The endogenous peroxidase activity was blocked using a 2.5% hydrogen peroxide solution. The tissue sections were subjected to heat-induced epitope retrieval by incubation in sodium citrate buffer (10 mM, pH 6.0) for 30 min in a 95 centigrade water bath, followed by blocking with 3% goat serum for 30 min. The primary antibody was then applied overnight, and the secondary antibody, biotin-goat anti-rabbit IgG (Zymed 65–6140, Invitrogen, CA, USA) was applied for 30 min, followed by the label antibody (ABC Elite, Vector Laboratories, Burlingame, CA, USA) for 30 min. The DAB chromagen (Dako Cytomation, Carpinteria, CA, USA) was applied for 3 min, followed by 2 rinses in distilled water. Mayers Hematoxylin (Histolab) was used as a counterstain.

### Statistical analysis

Statistical analyses were performed using Chi-squared test.

## Results and Discussion

In this study, we used the previously functional transgenic animal models, sm22α-rtTA [15] and tetop-LA^G608G^ [16], to develop a tissue-specific and inducible transgenic animal model for HGPS with expression in the vascular smooth muscle cells of the aorta to further the study the most lethal aspect of this disease.

**Figure 1:**
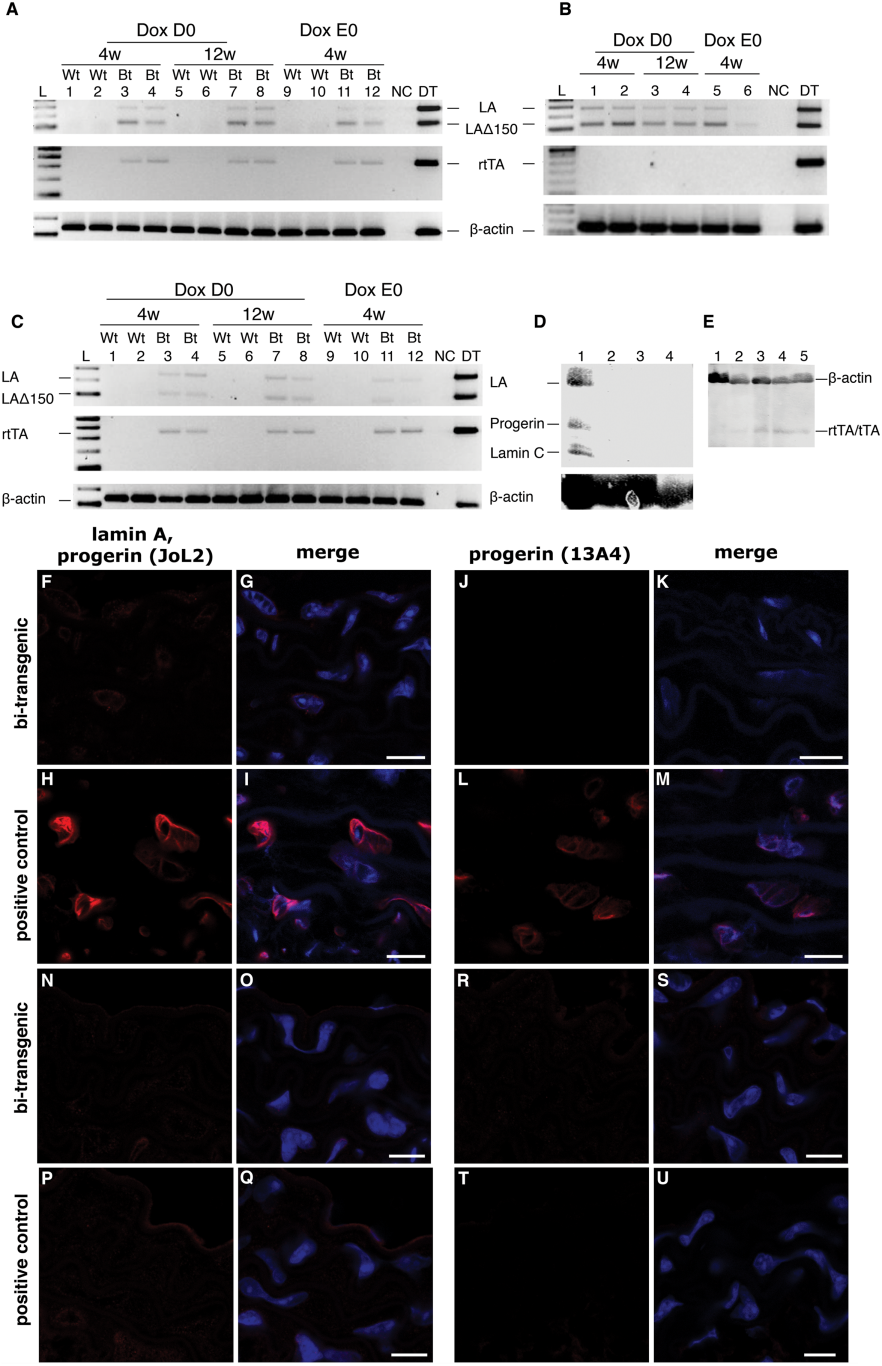
Low levels of transgene expression in the aortic arch. (A–C) RT-PCR using mRNA from the aortic arch showed very weak amplification products for human lamin A, lamin Adel150 and the reverse transactivator after 35 cycles of PCR. (A, C) RT-PCR on samples from bi-transgenic mice encoding both the reverse transactivator (sm22α-rtTA^+^) and the lamin A minigene (tetop-LA^G608G+^) that were supplied with doxycycline from the date of birth until postnatal week 4 (Dox D0, lanes 3–4) or 12 (Dox D0, lanes 7–8), or supplied with dox during embryogenesis and postnatally for 4 weeks (Dox E0, lanes 11–12). (A) C57BL/6J genetic background. (C) C57BL/6J; FVB/NCrl mixed genetic background. (B) RT-PCR for human lamin A and lamin Adel150 in samples from transactivator negative control animals (tetop-LA^G608G+^; sm22α-rtTA^−^, lanes 1–6) supplied with doxycycline from the date of birth until postnatal week 4 (Dox D0, lanes 1 and 2, C57BL/6J and C57BL/6J; FVB/NCrl genetic background, respectively) or week 12 (Dox D0, lanes 3 and 4, C57BL/6J and C57BL/6J; FVB/NCrl genetic background, respectively), or that were supplied with doxycycline during embryogenesis and postnatally for 4 weeks (Dox E0, lanes 5 and 6, C57BL/6J and C57BL/6J; FVB/NCrl genetic background, respectively). Bt, bi-transgenic. Wt, wild-type. NC, control with no template. DT, sample from a different transactivator strain was used as a control for the PCR assay and showed amplification for human lamin A and lamin Adel150 with cDNA from the bone of bi-transgenic tetop-LA^G608G+^; Sp7-tTA^+^ mice [18]. Genomic DNA from a tetop-LA^G608G+^; sm22α-rtTA^+^ bi-transgenic animal was used as a positive control for the amplification of the reverse transactivator (350 base pair product) [15]. The RT-PCR results for β-actin served as a control. (D) Western blot analysis on protein extracts from pooled aortic regions from bi-transgenic tetop-LA^G608G+^; sm22α-rtTA^+^ (lane 2) and tetop-LA^G608G+^; NSE-tTA^+^ (lane 3) animals did not show transgenic expression of human lamin A and progerin. A single transgenic tetop-LA^G608G−^; sm22α-rtTA^+^ animal carrying only the transactivator was used as a negative control (lane 4). Protein extract from HGPS patient cell line AG11513A was used as positive control (lane 1). (E) Western blot analysis on protein extracts from pooled aortic regions from bi-transgenic tetop-LA^G608G+^; sm22α-rtTA^+^ (lanes 2 and 5) and single transgenic animals, tetop-LA^G608G−^; NSE-tTA^+^ (lane 3) and tetop-LA^G608G−^; sm22α-rtTA^+^ (lane 4). Protein extracts from wild-type tissue, tetop-LA^G608G−^; NSE-tTA^−^, was used as a negative control (lane 1). (F–G and J–K) Very few transgene positive cells, <1%, were detected in the aortic arches of bi-transgenic animals at postnatal week 12 using antibodies specific for human lamin A/C and progerin (JoL2) (F–G), and human progerin (13A4) (J–K). (H–I and L–M) Positive staining was obtained using the same antibodies, on sections of the aortic arch from tetop-LA^G608G+^; NSE-tTA+ bi-transgenic mice [17]. (N–O and R–S) Very few transgene protein positive cells, <1%, were detected in the aortic arches of adult bi-transgenic animals, not supplied with doxycycline for the last 4 weeks prior to sacrifice, using antibodies for human lamin A/C and progerin (JoL2) (N–O), and human progerin (13A4) (R–S). (P–Q and T–U) Almost no positive staining was obtained using the same antibodies, on sections of the aortic arch from tetop-LA^G608G+^; NSE-tTA+ bi-transgenic mice supplied with doxycycline for 3 weeks (indicating a significant down-regulation of the transgenic expression with the doxycycline supplement). G, I, O, Q: merge of the transgenic lamin A and progerin with DAPI fluorescence signals. K, M, S, U: merge of the progerin and DAPI fluorescence signals. Scale bars: 10 µm.

Previously published results using sm22α-rtTA have shown that it could induce target transgene expression in the presence of doxycycline in the aortic arch, abdominal aorta and thoracic aorta, with the strongest induction observed in the aortic arch [15]. The aortic arch, thoracic aorta and abdominal aorta were isolated from bi-transgenic, control and wild-type animals, and the tissues were analyzed by RT-PCR, immunofluorescence and immunohistochemistry. Several conditions and time points were analyzed. To analyze the transgene expression at the RNA level, we used RT-PCR with primers that were specific for human lamin A, lamin Adel150 (progerin), and the reverse transactivator (Figures 1A–1C). Since the tetop-LA^G608G^ transgene contained a minigene with the human *LMNA* c.1824c>T; p.G608G mutation, which has previously been shown to generate only a partial activation of a cryptic splice site, we expected both wild-type lamin A and lamin Adel150 to be expressed from the transgene. However, the RT-PCR amplification of human lamin A and lamin Adel150 yielded very weak signals, even after 35 cycles of amplification (Figures 1A–1C). The weak signal was also observed in samples that did not carry the reverse transactivator transgene (Figure 1B), which suggested that the weak amplification product occurred due to the leakiness of the system, which has been reported in previous studies that used the tet-ON/OFF model systems [20]–[22]. A positive control reaction was performed using cDNA synthesized from RNA extracted from the bone of a previously reported animal model containing the tet-OFF system with the same lamin A target gene, available in the lab [18]. The positive control reaction yielded strong amplification of both the human lamin A and lamin Adel150 transcripts, confirming that the experimental procedure was functional (Figures 1A–1C). To analyze the expression of the reverse transactivator in the arteries, PCR with primers specific for the rtTA was performed on cDNA derived from samples from the bi-transgenic tetop-LA^G608G+^; sm22α-rtTA^+^ animals (Figures 1A, 1C). The RT-PCR confirmed the presence of the transactivator in the arteries but only resulted in a weak amplification signal. Western blot analysis on protein extracts from pooled aortic regions from bi-transgenic tetop-LA^G608G+^; sm22α-rtTA^+^ animals did not show transgenic expression of human lamin A and progerin (Figure 1D). Western blot experiments using an antibody to the transactivator (rtTA) showed a weak signal in protein extracts from the arteries of bi-transgenic animals and single transgenic animals for the sm22α-rtTA or the NSE-tTA (Figure 1E). One reason for the low expression of the transactivator could be the difference in genetic backgrounds since the original report of the sm22α-rtTA was based on offspring from sm22α-rtTA hybrids on C57BL/6 X CBA that were intercrossed with a target gene on C57BL/6 background [15]. However, this was unlikely because similarly weak amplification products were obtained using the aortic tissue samples extracted from animals with both pure C57BL/6J (Figure 1A) and mixed C57BL/6J; FVB/NCrl genetic backgrounds (Figure 1C). Similar weak amplification products were also observed using samples from mice that were administered doxycycline for different time periods, during embryogenesis to postnatal week 4, and from the date of birth to postnatal week 4 or 12 (see lanes 11–12, 3–4 and 7–8, respectively, in Figures 1A and 1C). Previous results have indicated that the tet-ON system is preferable to the tet-OFF system because induction by doxycycline is more rapid [12]. Thus, doxycycline addition, particularly during early/prenatal development, was expected to result in high transgene expression, which was not evident at the RNA level.

To monitor the expression of transgenic human lamin A and progerin at the protein level in the different aortic sections, we performed immunofluorescence staining using the anti-human-lamin A/C antibody (JoL2) which cross reacts with lamin A/C and progerin of human origin, but does not cross-react with mouse lamins (Figures 1F–1I). Unfortunately, only a few weakly positive cells, <1%, were found in samples from bi-transgenic animals (in both genetic backgrounds C57BL/6J and C57BL/6J; FVB/NCrl) that were supplied with doxycycline for 12 weeks after birth (data not shown). Aortic sections from bi-transgenic animals incubated with only the secondary antibody, used for transgene detection, did not show any fluorescence signal (data not shown). Aortic sections from wild-type littermate controls, stained with antibodies used for transgene detection, also showed no staining indicating that the weakly positive cells were exhibiting specific staining (data not shown). There were no positive cells in 4-week-old animals that were administered doxycycline treatment during embryogenesis or postnatally (data not shown). It is well known that with the progression of disease in HGPS, the smooth muscle cell population in arteries progressively decreases and the thickness of the vascular medial layer from the aortic arch to the abdominal aorta decreases [7], [9]. In a successful model system, we expected to have a high positive fluorescence signal for progerin and structural defects due to progerin toxicity, specifically in the aortic arch and the first part of thoracic aorta, compared to the abdominal aorta. However, in agreement with the results from the RT-PCR analysis and the immunofluorescence analysis using the transgene-specific lamin A/C antibody (JoL2), there were no progerin-positive immunofluorescent cells detected using an antibody against progerin (13A4) in any of the three aortic regions in bi-transgenic animals from all of the studied experimental groups (Figures 1J–1K, and data not shown). Aortic tissue from bi-transgenic animals with the same lamin A target gene driven by a different transactivator, NSE-tTA, was included as a positive control [17] (Figures 1D–E, 1H–I, 1L–M). The failure to detect human lamin A and progerin expression from aortic tissues with the NSE-tTA transactivator on Western blot (Figure 1D) (even though positive cells were seen on the immunofluorescence analysis using the transgene-specific lamin A/C and progerin antibodies (Figure 1H–I, 1L–M)) could be caused by heterogenous tissue and the fact that the protein extraction included cells from the aortic arch and all the way down to the abdominal aorta. Our previous analysis, on immunofluorescence from aortic regions of these control animals, have indicated a higher fraction of cells that express the transgene in the aortic arch compared to the thoraic aorta (data not shown). A control group of bi-transgenic animals that were not supplied with doxycycline were analyzed by immunofluorescent which showed a few positive cells, in the aortic arch sections, for the transgene-specific lamin A/C (Figure 1N–O) and progerin antibodies (Figure 1R–S), confirming the leakiness of the system. Treatment with doxycycline in the NSE-tTA+; tetop-LA^G608G+^ animal model the addition of doxycycline for three weeks was sufficient to downregulate the transgenic expression (Figure 1P–Q, 1T–U).

**Figure 2:**
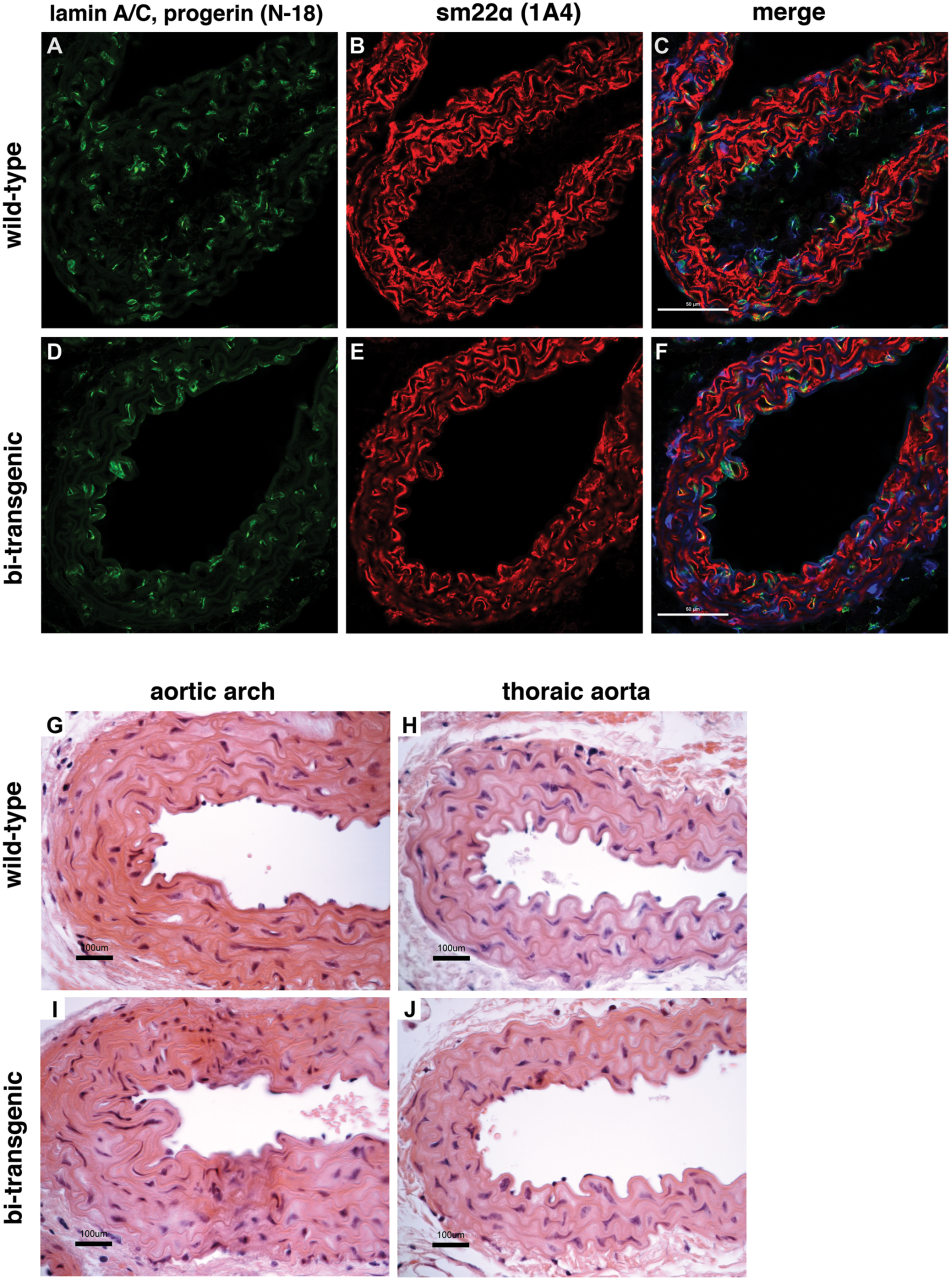
Expression of mouse lamin A/C and sm22α-actin is unaffected in the aortic arch. Immunofluorescence staining with an anti-human-lamin A/C antibody (N-18), which also binds to progerin of human origin and lamin A/C of mouse origin, and an antibody for vascular smooth muscle cells (1A4) in wild-type (A–C) and bi-transgenic tetop-LA^G608G+^; sm22α-rtTA^+^ animals (D–F). Representative images from the sections of aortic arches of mice with the C57BL/6J; FVB/NCrl genetic background supplied with doxycycline from the date of birth until postnatal week 12. Scale bars: 50 µm. C, F: merge of the lamin A/C, sm22α-actin, and DAPI fluorescence signals. (G–J) Histological examination of aortic sections with haematoxylin eosin staining shows normal structure of the aorta. G, I: aortic arch. H, J: thoraic aorta. Scale bars: 100 µm.

**Figure 3:**
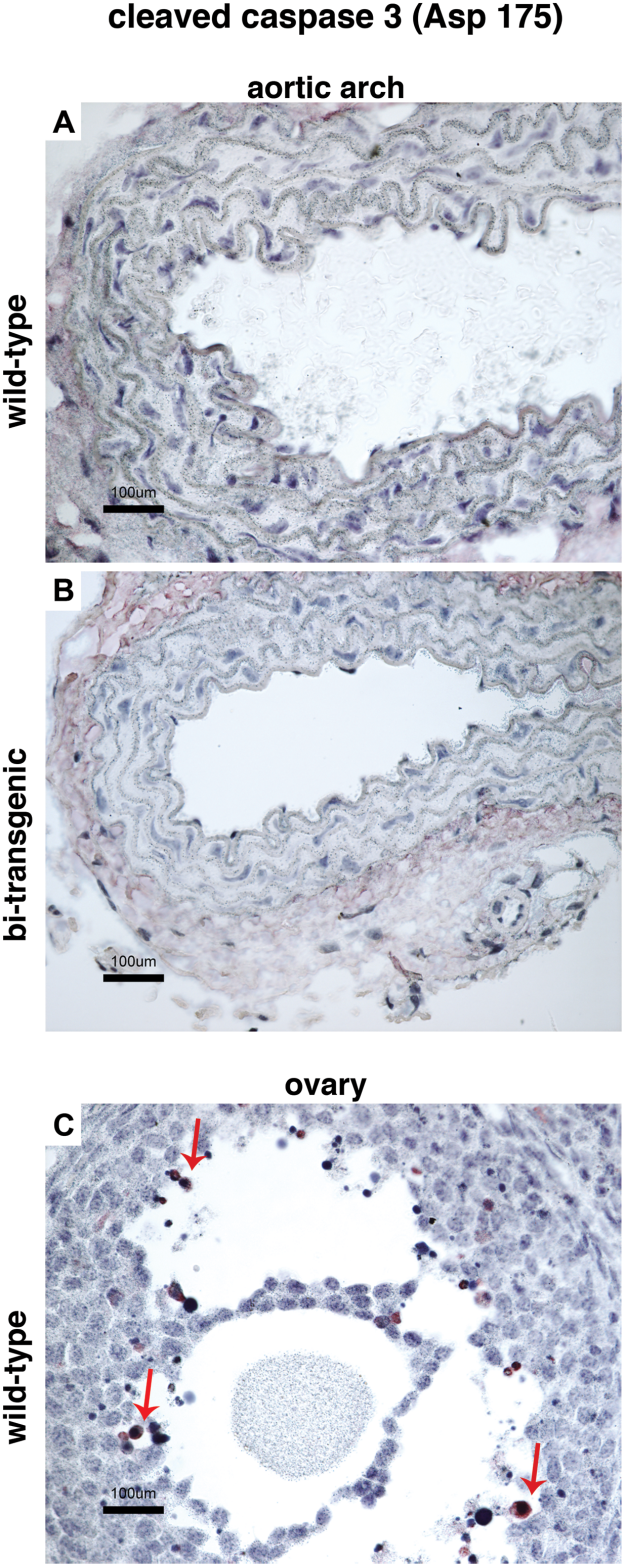
No signs of increased apoptosis in the vascular smooth muscle cells of the aortic arch. Immunohistochemical sections from the aortic arches of wild-type (A) and bi-transgenic tetop-LA^G608G+^; sm22α-rtTA^+^ (B) mice supplied with doxycycline from the date of birth to postnatal week 12, stained with an anti-Cleaved Caspase 3 antibody (Asp 175) to highlight apoptotic cells. (C) A section of the ovary from a wild-type animal used as a positive control tissue for apoptotic cells. Arrows indicate apoptotic cells. Scale bars: 100 µm.

To analyze whether the expression of transgenic human lamin A and progerin, despite being nearly undetectable at the RNA level at the time points analyzed, resulted in pathological changes such as the severe loss of vascular smooth muscle cells, we analyzed the expression of the lamin A and C proteins (with the N-18 antibody) in the vascular smooth muscle cells of the aortic arch, thoracic aorta and abdominal aorta (Figures 2A–2F and data not shown). Immunofluorescence analysis revealed a normal expression pattern of the A-type lamins (lamins A and C) in the vascular smooth muscle cells of bi-transgenic mice (Figures 2D–2F) similar to wild-type animals (Figures 2A–2C). In agreement with the immunofluorescence results, there were no signs of pathology in haematoxylin and eosin stained sections and no indication of loss of vascular smooth muscle cells in the different aortic regions (Figures 2G–2J, and data not shown). The expression of progerin in HGPS has previously been associated with increased apoptosis [23]. We therefore decided to investigate whether there was an increased population of apoptotic cells in the aortic arch, thoracic aorta, and abdominal aorta of bi-transgenic animals. However, immunohistochemistry using an antibody for cleaved caspase 3 (Asp 175) did not show increased apoptosis in the aortic sections from bi-transgenic animals when compared to wild-type animals (Figure 3).

Despite the absence of reports of toxicity associated with the administration of 2 mg/ml of doxycycline during embryogenesis, we carefully analyzed the numbers of individual genotypes for signs of embryonic lethality. Statistical analyses were performed to evaluate the deviation from Mendels law (see Table 1 for Genotype frequencies). Mendelian inheritance showed no significant deviation from expected values, (for pure C57BL6/J mice with doxycycline supplied during embyrogenesis (N = 79), P = 0.093 and for C57BL6/J and FVB/NCrl hybrids with doxycycline supplied during embryogenesis (N = 35), P = 0.219), for Hardy-Weinberg equilibrium based on genotype, when assessed with the Chi-squared test. Similarly no deviation from expected values from Mendels law was obtained for mice that were supplied with doxycycline postnatally (for pure C57BL6/J mice (N = 53), P = 0.059 and for C57BL6/J and FVB/NCrl hybrids (N = 48), P = 0.280).

**Table 1:** Genotype frequencies.

Doxycycline treatment	Background strain	Obtained genotype frequencies (%)			
		sm22α-rtTA^+^; tetop-LA^G608G−^	sm22α-rtTA^−^; tetop-LA^G608G+^	sm22α-rtTA^+^; tetop-LA^G608G+^	sm22α-rtTA^−^; tetop-LA^G608G−^
D0	C57BL/6J	30.2	26.4	34.0	9.4
	C57BL/6J; FVB/NCrl	14.6	29.2	33.3	22.9
E0	C57BL/6J	36.7	24.1	17.7	21.5
	C57BL/6J; FVB/NCrl	22.9	40.0	17.1	20.0

D0, mice supplied with doxycycline from the date of birth; E0, mice supplied with doxycycline during embryogenesis and postnatally; +, presence of transgene; −, absence of transgene. The expected frequency for each individual genotype was 25%.

Several investigators have reported problems using the tet-ON/OFF system and low expression of the transactivator [20]–[22], [24]. Unfortunately, we were unable to induce transgene expression of lamin A and progerin in the different regions of aorta using the sm22α-rtTA transactivator. The sm22α-rtTA has previously been used successfully by Mizrachi et al., wherein mice carrying the reverse transactivator-regulated promoter sm22α-rtTA expressed the target gene Ucp1 in the aortic arch, thoracic aorta and the abdominal aorta, with expression clearly detectable after 10 days of doxycycline treatment [15]. In our study, despite analyzing mice with different genetic backgrounds and allowing different time periods for doxycycline induction, we were unable to obtain expression of the HGPS allele above background levels. Currently, we can only speculate on the reasons for the lack of transgene expression. One possibility, is that epigenetic modifications of the promotor sequence of the sm22α-rtTA transgene may have arisen over multiple mouse generations, resulting in suppression of the rtTA expression [22], [25]–[26].
